# Parallel assessment of male reproductive function in workers and wild rats exposed to pesticides in banana plantations in Guadeloupe

**DOI:** 10.1186/1476-069X-7-40

**Published:** 2008-07-30

**Authors:** Luc Multigner, Philippe Kadhel, Michel Pascal, Farida Huc-Terki, Henri Kercret, Catherine Massart, Eustase Janky, Jacques Auger, Bernard Jégou

**Affiliations:** 1 Inserm, U625, Rennes, France; 2 Université Rennes 1, IFR 140, Rennes, France; 3 Service Gynécologie-Obstétrique, CHU, Guadeloupe; 4 Université des Antilles et de la Guyane, Guadeloupe; 5 INRA, Station SCRIBE, Équipe Gestion des Populations Invasives, Rennes, France; 6 CIST, Guadeloupe; 7 Unité d'Hormonologie, CHU Ponchaillou, Rennes, France; 8 Service d'Histologie-Embryologie et de Biologie de la Reproduction, CECOS, CHU Cochin, Paris, France

## Abstract

**Background:**

There is increasing evidence that reproductive abnormalities are increasing in frequency in both human population and among wild fauna. This increase is probably related to exposure to toxic contaminants in the environment. The use of sentinel species to raise alarms relating to human reproductive health has been strongly recommended. However, no simultaneous studies at the same site have been carried out in recent decades to evaluate the utility of wild animals for monitoring human reproductive disorders. We carried out a joint study in Guadeloupe assessing the reproductive function of workers exposed to pesticides in banana plantations and of male wild rats living in these plantations.

**Methods:**

A cross-sectional study was performed to assess semen quality and reproductive hormones in banana workers and in men working in non-agricultural sectors. These reproductive parameters were also assessed in wild rats captured in the plantations and were compared with those in rats from areas not directly polluted by humans.

**Results:**

No significant difference in sperm characteristics and/or hormones was found between workers exposed and not exposed to pesticide. By contrast, rats captured in the banana plantations had lower testosterone levels and gonadosomatic indices than control rats.

**Conclusion:**

Wild rats seem to be more sensitive than humans to the effects of pesticide exposure on reproductive health. We conclude that the concept of sentinel species must be carefully validated as the actual nature of exposure may varies between human and wild species as well as the vulnerable time period of exposure and various ecological factors.

## Background

Over the last 20 years, a growing number of observations worldwide have warned of increases in the frequency of reproductive abnormalities in wild male vertebrates [1]. Increases in the frequency of several modifications to male reproductive function have also been reported in humans [1-4]. These include an apparent decline in semen quality and an increase in the incidence of both testicular cancer and congenital abnormalities, such as cryptorchidism and hypospadia. The reasons for these changes are unclear. The short time period over which they have occurred suggests a role for environmental rather than genetic factors. In recent decades, both animals and humans have been subjected to considerable changes in physical, chemical and biological aspects of the environment. Over the same period, sociocultural factors (e.g. diet and lifestyle) affecting humans have also changed considerably. The observed reproductive phenomena probably have multifactorial causes, but attention has principally focused on chemical agents. Chemical pollution has frequently been associated with reproductive effects in wildlife [5,6] and recent epidemiologic and occupational studies have also identified a possible syndromic role of chemical compounds [7,8]. The similarities and differences in reproductive abnormalities observed in humans and wildlife have stimulated fruitful interactions between toxicologists, epidemiologists, clinicians, ecotoxicologists and zoologists [4,9].

Humans and wildlife are exposed to a number of pesticides in the environment. Many of these compounds have been shown to function as endocrine disruptors [10,11]. Other pesticides not formally identified as endocrine disruptors, such as dibromochloropropane (DBCP), may also damage the testes and spermatozoa directly or indirectly [12]. Nevertheless, epidemiologic and experimental studies in the field, particularly as concerns environmental exposure, have provided conflicting results [7]. The use of joint ecoepidemiologic and human epidemiologic surveys for the simultaneous collection and comparison of data is strongly recommended [13]. However, to our knowledge, no such study has yet been undertaken. As a consequence, the notion that wildlife species may be used as sentinel species for human syndromes remains largely theoretical and/or speculative.

In this study, we jointly assessed – during the same time period and at the same site – various endpoints of male reproductive function in humans and wild mammals, both chronically exposed to pesticides. This study was carried out in Guadeloupe (an island in the French West Indies), which has areas of intensive agriculture using large amounts of pesticides, particularly on banana plantations. We studied male employees working in banana plantations and male rats living in the banana fields. Comparisons were made with men working in non-agricultural sectors and rats living in an area devoid of direct human sources of pollution.

## Materials and methods

### Study area

Guadeloupe consists of two main islands, Grande Terre and Basse Terre, separated by a narrow sea channel. Agriculture is the main economic activity and banana is the principal crop. The banana plantations are mostly located on the rainy slopes of Basse-Terre and intensive pesticide use controls insects, nematodes and fungi in these plantations. The National Park of Guadeloupe encompasses the central part of Basse-Terre and includes the Natural Reserve of Grand Cul-de-Sac Marin. While we are aware that no area can be totally pollution-free, this park has historically been protected from direct pollution by humans and provides an opportunity for research on wildlife less strongly affected by pollution.

### Human study

The study was carried out from 1999 to 2001. The study population consisted of 20- to 50-year-old male workers from Basse-Terre regularly followed up by the Guadeloupe Occupational Health Service. Information meetings were organized in the field, at which the men were informed that the study protocol included the collection of a semen sample, a blood sample, and physical and andrological examinations. Basic data, such as age and current job were collected for all men, including those who later declined participation in other aspects of the study. Each participating worker was given an appointment at the University Hospital of Pointe à Pitre. Blood samples were collected and medical examinations carried out, and information about occupational history and past and present exposure to chemical and physical agents was collected in face-to-face interviews. An indemnity of 80 Euros was paid to each participant who gave written informed consent and the Ethics Committee of Guadeloupe approved this study.

During the hospital visit, subjects underwent a physical and andrological examination, performed by a single physician unaware of the subject's exposure status. A detailed examination of external genitalia and staging of secondary sexual development were carried out according to WHO recommendations [14]. Testicular volumes were evaluated using the Lambert formula: volume (cm^3^) = 0.71 × major axis (cm) × minor axis (cm)^2^. Abnormalities in testis thermoregulation are known to alter semen characteristics, so scrotal temperatures were measured in naked subjects in the supine position [15]. Demographic data, medical, surgical, urogenital and reproductive history, recent illnesses, fever and treatment and lifestyle data were collected using a standardized questionnaire during a face-to-face interview with the physician.

Pesticide exposure status was assessed with a questionnaire. Information was collected yearly concerning occupational and non occupational exposure to chemical substances (pesticides, solvents, metals and diesel exhaust fumes) or physical agents (radiant heat and ionizing radiation). The number of years of use and annual frequency of use was recorded for each category of chemical substance or physical agent. We asked about whether pesticides were applied in a banana plantation, sugar cane plantation or other agricultural setting, and distinguished between the agricultural use of pesticides and kitchen garden activity or other domestic exposure. One occupational physician specifically trained in agricultural activities assessed job descriptions blindly, to assess the degree of correlation between job tasks and declared occupational exposures.

Semen samples were collected by masturbation in the hospital after a period of sexual abstinence for 2 to 7 days. Semen analysis was performed according to WHO guidelines [16], at the Pathology Laboratory of the University Hospital of Pointe à Pitre. Semen samples were analyzed by 2 technicians blind to exposure status and specifically trained on site. Intra- and inter-technician variations in the assessment of sperm concentration and motility were evaluated several times before and during the study and were considered satisfactory, the coefficients of variation being typically around 5 to 10%. Sperm morphology was assessed from smears after Shorr staining, using the modified David classification [17], by a single technician at the Reproductive Biology Laboratory, Cochin University Hospital, Paris.

Blood samples were collected between 8 and 9 a.m. and serum samples were stored at -20°C until the analysis. FSH (follicle-stimulating hormone) and LH (luteinizing hormone) levels were determined by immunoradiometric assay (Immunotech, Marseilles, France). The dose-response curve of FSH was calibrated against the WHO international standard of 94/632, and the LH dose-response curve was calibrated against the 2^nd ^IS international standard of 80/552. Total testosterone concentration was measured by radioimmunoassay (Immunotech, Marseilles, France) and dimeric inhibin B concentration was assessed by enzyme-linked immunosorbent assay (Oxford Innovation Ltd, Oxford, UK). Results were expressed as concentrations for testosterone (ng/ml), for inhibin B (pg/ml) and for FSH and LH (IU/l). Between- and within-assay coefficients of variation were < 8 % for FSH and LH, < 11 % for testosterone and < 17 % for inhibin B.

We analyzed data for age, weight, height, body mass index, smoking habits (past or current smoker *vs*. non smokers), alcohol consumption (current drinker *vs*. non drinker), recent illness (yes *vs*. no), fever or medical treatment during the past 3 months (yes *vs*. no), fertility status scored as at least one live-born child (yes *vs*. no) and, for fertile participants, the total number of children and time to pregnancy for the most recent child, days of sexual abstinence before semen analysis, testicular volume and scrotal temperature. In addition, the month in which the semen analysis was performed was noted, to account for a possible seasonal effect. The categories spring/summer *vs*. autumn/winter were compared and, as there are no significant differences in temperature or day length between seasons on Guadeloupe, we also considered summer/autumn (wet season) *vs*. winter/spring (dry season). The semen characteristics and hormones studied were: pH, semen volume (ml), sperm concentration (millions/ml), total sperm count (millions, the product of semen volume and sperm concentration), sperm viability (the percentage of live spermatozoa), "a"-type progressive motility (the percentage of rapid and progressive spermatozoa), "a"+"b"-type progressive motility (the percentage of rapid and slow progressive spermatozoa), sperm morphology (the percentage of morphologically normal spermatozoa) and the multiple anomaly index (mean number of morphological defects per abnormal spermatozoa). Semen characteristics were also categorized as dichotomous variables, using WHO reference values [16] as thresholds: 2 ml for seminal volume, 20 millions/ml for sperm concentration, 40 millions for total sperm count, 25% for "a"-type progressive motility and 50% for "a" + "b"-type progressive motility. The threshold selected for sperm morphology was the median percentage of morphologically normal spermatozoa in non exposed men (13%).

### Animal study

We selected two sites extremely different in terms of agricultural activity. The "pesticide-exposed area", located in Neuf-Chateau (south-east Basse-Terre), has been exclusively devoted to banana cultivation since the mid-20th Century. The "pesticide-free area" was Fajou Islet in the Grand Cul-de-Sac Marin lagoon, within the marine reserve of the National Park.

Trapping and field operations took place between the 18th and the 28th of January 2000, a period of high reproductive activity in French West Indies rodent populations. Ship rats (*Rattus rattus*), Norwegian rats (*Rattus norvegicus*) and Javanese mongoose (*Herpestes auropunctatus*), all alien species in the French West Indies [18], were trapped using Manufrance^© ^live traps baited with a mixture of peanut butter, oat flakes and sardine oil [19]. The trapped animals were killed in the field by intrathoracic pentobarbital injection. Blood was collected immediately by heart puncture. Animals and blood samples were stored in a container at + 4°C and transferred within 45 minutes to a laboratory specifically installed for this study.

Mammals were sexed and males selected, weighed and dissected. The testes, epididymis, ventral prostate and seminal vesicles were removed and weighed. One testis per animal was fixed by immersion in Bouin's solution for histology, the second testis and one epididymis being immediately frozen at – 20°C. Both eye balls were removed and stored in 10% formaldehyde at room temperature for more than 3 months to tan eye lenses. Eye lenses were cleaned and dried at 105°C until their weight stabilized. Lens pair weight was used as a surrogate for age [20]. Each fixed testis was embedded in paraffin, and 5 μm sections were cut, stained with hematoxylin and eosin and examined under a light microscope [21]. The number of sperm in the testis and caudal epididymis was assessed as previously described [22]. The total number of sperm was defined as the sum of testicular and epididymal sperm reserves. The gonadosomatic index was calculated as (testis weight/body weight) × 100.

Serum fractions were stored at -20°C until use. Rat FSH levels were determined by immunoradiometric assay (BiocodeHycel, Belgium) and rat LH levels, by radioimmunoassay (BiocodeHycel, Belgium). Dose-response curves for FSH and LH were calibrated against the corresponding NIH standard. Total rat testosterone levels were determined by radioimmunoassay (Immunotech, Marseilles, France). Results were expressed as concentrations (ng/ml for testosterone, IU/l for FSH and LH). The intra- and inter-assay coefficients of variation were < 6, 7 and 8 % for testosterone, FSH and LH, respectively.

### Statistical analysis

We used chi-squared or Fisher's exact tests for categorical variables and unpaired t-tests (equal variances) and analysis of variance/covariance for continuous variables, in both human and animal studies. We normalized distributions by applying a log10 transformation or a square-root transformation and then used the Kolmogorov-Smirnov test to check that the resulting data were normally distributed. The effect of exposure on the categorized human seminal outcomes was assessed, taking possible confounding factors into account, by logistic regression analysis to produce odds ratios (OR) and 95 percent confidence intervals (95% CI). Factors were considered confounding if their p values in univariate analysis were below 0.2, and were included in the final model if they modified the odds ratio of the outcome by more than 10 percent [23]. The potential confounding variables evaluated were age, alcohol and tobacco consumption, solvent exposure, past history of genital infection, sexual abstinence, and season of semen analysis. Age and duration of abstinence were systematically included as known confounding factors for semen variables. Linear regression was used to assess the relationship between eye lens weight, body weight and total sperm reserve in the animal study. All analyses were carried out using the Statview software package (SAS Institute Inc., Cary, USA). All tests were two-sided, and the results were considered significant if P < 0.05.

## Results

### Human Study

In total, 101 of the 594 men approached for this study (17%) agreed to participate. There was no difference in age or proportion of banana plantation workers and workers not employed by banana plantations between those agreeing to and declining participation (data not shown). Six men who agreed to participate were excluded for one of the following reasons: past chemotherapy for Hodgkins disease, current treatment for infertility, orchidectomy, current active genital infection or sexual abstinence for less than 48 hours before semen analysis. Forty-two were employed as banana plantation workers and regularly used pesticides as part of their job. They worked on 13 plantations but reported having applied pesticides in 37 different plantations over the course of their careers, corresponding to more than 80% of the local area under banana. We listed the chemical products used in the banana plantations from the notebooks used to record pesticide applications. The pesticides in use at the time of the study belonged to the organophosporus (cadusaphos, ethoprophos, isazophos, pyrimiphos-ethyl, terbufos) and carbamate (aldicarb) classes. The median duration of pesticide use was 14 years for banana workers. The mean annual frequency of application was 30 days, and was similar for all banana plantation workers. A group of 45 manual and office workers enrolled in the study did not apply pesticides in either occupational or non occupational circumstances. Finally, eight men were in contact with pesticides under miscellaneous conditions, including occupational exposure in sugarcane fields or vegetable root plots or domestic applications in kitchen gardens. This group was not analyzed further due to the small number of subjects and their heterogeneity in terms of the chemical family of pesticides used and the frequency and intensity of application.

Table 1 presents the general characteristics of the 87 participants included in the final analysis and the comparisons between exposure groups. Banana plantation workers were younger than unexposed men. The proportion of smokers and men exposed to solvents was significantly higher in the unexposed group than among banana plantation workers. Likewise the proportion of drinkers was slightly (not significantly) higher in unexposed men.

**Table 1:** General, lifestyle and occupational data for men

**Characteristics**	**Non exposed** **workers** ** (n = 45)**	**Pesticide-exposed** **banana plantation** **workers** ** (n = 42)**	**P**
Age, *mean (SD)*	38.4 (7.6)	34.8 (6.5)	0.02 *
Body mass index, *mean (SD)*	24.6 (3.3)	24.6 (3.3)	0.97 *
Body mass index, *n (%)*			
< 25	28 (62)	26 (62)	0.86†
25 – < 30	15 (33)	13(31)	
> 30	2 (5)	3 (7)	
Tobacco, *n (%)*			
Non smoker	23 (51)	32 (76)	0.02†
Current or former smoker	22 (49)	10 (24)	
Alcohol, *n (%)*			
Non drinker	10 (22)	15 (36)	0.17†
Current or former drinker	35 (78)	27 (64)	
Drugs, *n (%)*			
Non consumers	41 (91)	37 (88)	0.64†
Current or former consumers	4 (9)	5 (12)	
Heat exposure, *n (%)*			
No	43 (96)	38 (90)	0.35†
Yes	2 (4)	4 (10)	
Solvent exposure, *n (%)*			
No	28 (62)	36 (86)	0.01†
Yes	17 (38)	6 (14)	
Diesel exhaust exposure, *n (%)*			
No	38 (84)	36 (86)	0.87†
Yes	7 (16)	6 (14)	
Season of semen analysis, *n (%)*			
Spring or summer	28 (62)	20 (48)	0.17†
Autumn or winter	17 (38)	22 (52)	
Dry season	23 (51)	30 (71)	0.05†
Wet season	22 (49)	12 (29)	
Abstinence before semen analysis (days), *mean (SD) mean (SD)*	4.1 (1.3)	3.9 (1.0)	0.80 *

*: unpaired t-test; †: χ^2 ^test

A history of genital infection was more frequent among banana workers than among unexposed men (table 2). Similarly, a higher proportion of banana plantation workers than of unexposed men had conceived a child. No difference was observed between the two groups in time to pregnancy for the last conception. No significant difference between exposed and unexposed workers was found for any of semen characteristic or hormone level (Table 3). Although not significant after adjustment for covariables, sperm concentration and total sperm count were 20% lower in the pesticide-exposed banana plantation workers than in workers not exposed to pesticides. Based on WHO semen reference values, used as thresholds, banana plantation workers were found to have a slightly, but not significantly higher OR only for a low total sperm count (OR: 2.2, 95% CI 0.5 – 10.4). Following stratification for the number of years of pesticide application, the association between a low sperm concentration or total sperm count and having been exposed for more than 14 years became stronger, but remained non significant (Table 4).

**Table 2:** Medical, andrological and reproductive characteristics in men

**Characteristics**	**Non exposed** **workers** ** (n = 45)**	**Pesticide-exposed** **banana plantation** **workers** ** (n = 42)**	**P**
Tanner, *n (%)*			
3	2 (5)	1 (2)	0.75†
4	15 (33)	12 (29)	
5	28 (62)	29 (69)	
Testicular volume (ml), *mean (SD)*			
Right testis	20.3 (8.3)	20.3 (8.7)	0.99*
Left testis	18.1 (7.7)	18.8 (6.4)	0.65*
Both testes	38.4 (15.3)	39.1 (12.6)	0.82*
Testicular temperature (°C), *mean (SD)*			
Right testis	35.2 (0.7)	35.1 (0.7)	0.53*
Left testis	35.3 (0.7)	35.2 (0.6)	0.78*
Central temperature (°C), *mean (SD)*	36.9 (0.4)	36.9 (0.3)	0.76*
Past genital infection, *n (%)*			
No	40 (89)	28 (67)	0.01†
Yes	5 (11)	14 (33)	
Inguinal hernia (treated), *n (%)*			
No	43 (96)	39 (93)	0.59†
Yes	2 (4)	3 (7)	
Has conceived at least one child, *n (%)*			
No	16 (36)	7 (17)	0.06†
Yes	29 (64)	35 (83)	
Time to pregnancy (last conception), *n (%)*			
≤ 12 months	26 (96)	28 (90)	0.37†
> 12 months	1 (4)	3 (10)	

*: unpaired t-test; †: χ^2 ^test

**Table 3:** Semen characteristics and serum hormone levels in men

**Characteristics**	**Non exposed** **workers** ** (n = 45)**	**Pesticide-exposed** **banana plantation** **workers** ** (n = 42)**	**P**
**Semen characteristics**			
Volume of the ejaculate (mL), *mean (SD)*	3.8 (1.7)	3.4 (1.7)	0.44*
pH, *mean (SD)*	7.9 (0.2)	7.9 (0.2)	0.65*
Sperm concentration (millions/mL), *mean (SD)*	89.8 (81.1)	70.3 (59.4)	0.27*
Total sperm count (millions), *mean (SD)*	308 (236)	231 (229)	0.19*
Rapid and progressive sperm motility (%), *mean (SD)*	15.2 (8.6)	15.0 (6.8)	0.82*
Progressive sperm motility (%) *mean (SD)*	42.4 (11.9)	42.7 (13.9)	0.62*
Morphologically normal sperm (%), *mean (SD)*	13.9 (7.4)	13.0 (8.0)	0.36*
Multiple anomaly index, *mean (SD)*	1.71 (0.25)	1.73 (0.29)	0.79*
Live sperm (%), *mean (SD)*	54.1 (13.4)	54.5 (17.3)	0.56*
**Serum hormones**			
FSH (IU/L), *mean (SD)*	6.3 (4.4)	5.9 (4.1)	0.81†
LH (IU/L), *mean (SD)*	4.7 (1.9)	5.5 (2.6)	0.16†
Testosterone (ng/mL), *mean (SD)*	7.5 (2.5)	6.8 (1.7)	0.33†
Inhibin B (pg/mL), *mean (SD)*	170 (72)	168 (67)	0.63†

* adjusted for age, tobacco use, solvents, genital infections, season of sperm analysis and sexual abstinence.

† adjusted for age and body mass index

**Table 4:** Associations between semen characteristics and pesticide exposure in men

**Semen Characteristics**	**Non** **exposed** **workers** ** (n = 45)**	**Pesticide-exposed** **banana plantation** **workers** **(< 14 years)** ** (n = 21)**		**Pesticide-exposed** **banana plantation** **workers** **(> 14 years)** ** (n = 21)**	
	OR	OR (95%CI) *	P	OR (95%CI) *	P

Semen volume (< 2 mL)	1.0	1.0 (0.2, 6.0)	0.99	1.4 (0.2, 11.3)	0.77
Sperm concentration (< 20 millions/mL)	1.0	0.9 (0.2, 5.4)	0.92	2.0 (0.4, 10.1)	0.40
Total sperm count (< 40. millions)	1.0	1.7 (0.2, 12.1)	0.58	3.2 (0.5, 22.0)	0.24
"a" progressive sperm motility (< 25%)	1.0	1.6 (0.2, 17.4)	0.68	0.6 (0.1, 5.6)	0.59
"a" + "b" progressive sperm motility (< 50%)	1.0	0.7 (0.2, 2.3)	0.60	1.0 (0.3, 3.6)	0.99
Sperm morphology (< 13%)	1.0	0.9 (0.3, 2.8)	0.86	2.0 (0.6, 6.9)	0.28

* adjusted for age, tobacco use, solvents, genital infections, season of sperm analysis and sexual abstinence

### Animal Study

In total, 120 mammals were trapped: 75 in banana plantations and 45 in Fajou Islet. The very small number of trapped mongooses (n = 9) and the smaller numbers of Norwegian rats in Fajou Islet than in banana plantations (n = 19) ruled out the further analysis of these species. We captured 92 ship rats, and the proportion of males and females did not differ significantly between the sites (p = 0.29). The relationships between eye lens weight (a surrogate of age), body weight and sperm reserve were used to distinguish sexually mature from immature male ship rats. Five animals with a body weight less than 100 g (the cut-off generally used to distinguish between adult and non adult ship rats) with no epididymal and testicular spermatozoa had a dry eye lens weight below 20 mg. Three other animals with an absence of epididymal spermatozoa but with testicular spermatozoa had eye lens weights below 21 mg. For these eight rats, testis histology indicated that there was no, or only focal, spermatogenesis. We therefore used a threshold eye lens weight of 21 mg w to distinguish sexually mature ship rats from immature ones.

No significant difference was observed in the mean eye lens weight of ship rats captured in the two areas (table 5). This suggests that age structures of the two populations were similar. However, ship rats captured in banana plantations were significantly heavier than those captured in Fajou Islet. None of the absolute weights of reproductive organs differed significantly between ship rats from the two sites. However, the testis weight/body weight ratio, known as the gonadosomatic index, was lower in ship rats trapped in the banana plantations than for those trapped in Fajou Islet. This difference could not be accounted for by a difference in the "relative age" of the animals, as eye lens weight was included as a covariate. Serum testosterone levels were also significantly lower in ship rats trapped in banana plantations than in those trapped in Fajou Islet, whereas gonadotropin levels were similar in rats from both sites.

**Table 5:** General and reproductive characteristics in male ship rats

**Characteristics**	**Pesticide-exposed area** **(banana plantations)** **(n = 21)** ** mean (SD)**	**Pesticide-free area ** **(Fajou Islet)** **(n = 20)** ** mean (SD)**	**P**
Eye lens weight (mg)	29.4 (5.9)	29.0 (4.0)	0.92 †
Body weight (g)	218 (31)	166 (30)	< 0.0001 †
Testis weight (g)	3.68 (0.72)	3.47 (0.36)	0.07 *
Epididymal weight (g)	1.22 (0.5)	1.28 (0.2)	0.39 *
Prostate weight (g)	0.21 (0.11)	0.20 (0.06)	0.79 *
Seminal vesicle weight (g)	0.82 (0.63)	0.88 (0.31)	0.50 *
Testicular sperm reserves (millions)	437(141)	419 (73)	0.57 *
Epididymal sperm reserves (millions)	427 (304)	477 (167)	0.30 *
Total sperm reserves (millions)	864 (413)	896 (223)	0.56 *
Gonadosomatic index	1.70 (0.31)	2.13 (0.29)	< 0.0001 *
Testosterone (ng/mL)	11.2 (6.1)	19.1 (9.8)	0.02 *
LH (IU/L)	0.53 (0.46)	0.50 (0.18)	0.82 *
FSH (IU/L)	17.6 (3.6)	18.4 (6.3)	0.65 *

†: unpaired t-test;

*: adjusted for eye lens weight as a surrogate for age

## Discussion

There is widespread concern that reproductive function, particularly in males, is currently subject to the deleterious effects of chemical agents in the environment. According to the endocrine disruption hypothesis, exposure to chemicals, including pesticides, results in a range of reproductive disturbances in humans and in various wildlife species, some of which may be used as sentinel species indicating an increase in the threat to human reproduction and health [1,4,9,13].

Past chemical hazards to male reproductive function were revealed in a dramatic manner, in a study showing that banana plantation workers exposed to the nematocide DBCP had severely impaired spermatogenesis, leading to permanent infertility in some cases [12]. Other pesticides used in banana crops, such as chlordecone and ethylene dibromide, have also clearly been shown to cause spermatogenic damage in a laboratory setting [24,25].

The pesticides in use in banana plantations in Guadeloupe at the time of this study included five organophosphorous agents – cadusafos, ethoprophos, isazophos, pyrimiphos-ethyl and terbufos – and one carbamate derivative, aldicarb. To our knowledge, until this study, no epidemiological study had investigated the effects of these pesticides on male fertility.

Human semen samples are generally difficult to obtain, with participation rates generally below 20% [26]. The participation rate of 17% in our study is consistent with this observation. Our protocol allowed us to exclude all subjects with identified infertility risk factors [27] and to identify an occupationally exposed group that had applied pesticides and been continuously exposed to these agents over several years. While intrinsically restricted by the expected limitations of cross-sectional sperm studies and the small size of this study, our results suggest that exposure to the pesticides currently used in the banana plantations of Guadeloupe is not markedly detrimental to male fertility.

In Guadeloupe, ship rats are a commensal species, probably introduced by Spanish explorers before 1635 [18]. Age determination is a key element of population dynamics studies in wild mammals of this kind [28]. It has been shown that, for species with a life expectancy in the wild of less than one year (as in rats), eye lens dry weight is a suitable surrogate variable for the age of the animal [29]. Following the adjustment of values to account for differences in age between rats captured in pesticide-exposed and unexposed areas, the only parameter significantly affected was blood testosterone concentration. The observed decrease in circulating testosterone concentration may reflect an anti-androgenic effect of the substances polluting the banana fields. Indeed, a number of organophosphorus insecticides, including chlorphyriphos-methyl, malathion and diazinon, have been shown in toxicological experiments to inhibit testosterone synthesis/production [30-32]. It has also been suggested that aldicarb, one of the pesticides in use at the time of the study, acts in synergy with other pesticides as an endocrine disruptor in laboratory rats [33]. Furthermore, a toxic interaction between testosterone and aldicarb has been reported in the Japanese medaka fish [34]. However, the lack of reproductive toxicity studies on the organophosphorous pesticides used in Guadeloupe, and past contamination of banana plantations with organochlorine pesticides make it difficult to identify precisely the causal agents reducing blood testosterone levels. The affected rats displayed no significant change in LH concentration, possibly due to the fatness of rats trapped in banana plantations, which were more than 20 % heavier than Fajou Islet rats. This situation may have led to a number of metabolic changes, including, for example, an increase in circulating estradiol levels. However, this hypothesis remains to be confirmed experimentally. This difference in body weight reflects differences in food availability between the two habitats, as autopsy showed that ship rats do eat bananas. Due to this discrepancy in body weight, the gonadosomatic index was significantly higher for rats from the unexposed area than for rats from the pesticide-exposed area.

Several factors, such as the nature of the chemicals to which humans and animals were exposed and major differences in the generation time of humans and rats, must be taken into account when comparing the impact of pesticides on human and rat populations. For example, DBCP was scarcely used in the French West Indies in the past, but chlordecone was in widespread use until 1993 [35]. These chemicals were replaced by organophosphorus and carbamate insecticides. Thus, unlike humans, rats, with their much shorter generation time, have been exposed only to the most recently used pesticides. Other considerations to be borne in mind when comparing the human and rat populations is that rat populations are strictly territorial and therefore remain permanently in their habitat and that the periods of development (e.g., prenatally, adulthood) at which they are exposed may differ.

This study constitutes a prototype experiment. Major logistic efforts were made: a thorough historical analysis of environmental and agricultural conditions in Guadeloupe, the setting up of a spermatology laboratory, with the adequate training of local health technicians for human sperm handling, processing and analysis, the setting up of an animal autopsy laboratory, fully equipped for the manipulation and storage of biological samples and tissues and the coordination of several institutions for the capture of wild animals and of researchers from different disciplines including andrologists, wildlife specialists, biologists and epidemiologists. For the further development of such approaches, exposure measurements in both humans and animals, larger numbers of human volunteers and the inclusion of several wildlife species would all be relevant objectives. Furthermore, the relative impact of ecological factors on reproductive success in wildlife species should also be considered. These factors include habitat restriction, stress due to the intrusion of humans and changes in food supply. These, and subtle genetic differences in the population structure, could not be evaluated here.

## Conclusion

Wild rats seem to be more sensitive than humans to high levels of pesticides (or of other unidentified factors) in the environment. The concept of sentinel species is not as simple as it may appear. Understanding the very complex interactions between the plethora of potential contaminants and the organisms living in contaminated environments will require experiments including many parameters and should, ideally, span the life cycle of some of the species concerned.

## Abbreviations

DBCP: Dibromochloropropane; FSH: Follicle stimulating hormone; LH: Luteinizing hormone; WHO: World Health Organization.

## Competing interests

The authors declare that they have no competing interests.

## Authors' contributions

LM, PK, MP, JA and BJ designed the study, supervised the overall project and were involved in the interpretation of the results and revision of the paper. LM was responsible for the overall running of the study and did the statistical analysis. PK, FHT and EJ were responsible for recruiting the human subjects and for medical data collection. LM, PK, MP, JA and BJ were involved in the field study. HK and CM were involved in hormonal analysis. All authors read and approved the final manuscript. LM and PK equally contributed to this work.

## References

[B1] Toppari J, Larsen J, Christiansen P, Giwercman A, Grandjean P, Guillette L, Jégou B, Jensen TK, Jouannet P, Keiding N, Leffers H, McLachlan JA, Meyer O, Müller J, Rajpert-De Meyts E, Scheike T, Sharpe R, Sumpter J, Skakkebaek NE (1996). Male reproductive health and environmental xenoestrogens. Environ Health Perspect.

[B2] Carlsen E, Giwercman A, Keiding N, Skakkebaek NE (1992). Evidence for decreasing quality of semen during past 50 years. BMJ.

[B3] Auger J, Kunstmann JM, Czyglik F, Jouannet P (1995). Decline in semen quality among fertile men in Paris during the past 20 years. N Engl J Med.

[B4] Jégou B, Auger J, Multigner L, Pineau C, Thonneau P, Spira A, Jouannet P, Gagnon C (1999). The saga of the sperm count decrease in humans wild and farm animals. The Male Gamete: From Basic Science to Clinical Applications.

[B5] Colborn T, vom Saal FS, Soto AM (1993). Developmental effects of endocrine-disrupting chemicals in wildlife and humans. Environ Health Perspect.

[B6] Guillette LJ, Gunderson MP (2001). Alterations in development of reproductive and endocrine systems of wildlife populations exposed to endocrine-disrupting contaminants. Reproduction.

[B7] Swan SH (2006). Does our environment affect our fertility? Some examples to help reframe the question. Semin Reprod Med.

[B8] Olea N, Fernandez MF (2007). Chemicals in the environment and human male fertility. Occup Environ Med.

[B9] Guillette LJ (2006). Endocrine disrupting contaminants: beyond the dogma. Environ Health Perspect.

[B10] Sharpe R, Skakkebaek N (1993). Are estrogens involved in falling sperm counts and disorders of the male reproductive tract?. Lancet.

[B11] McLachlan JA (2001). Environmental signaling: what embryos and evolution teach us about endocrine disrupting chemicals. Endocr Rev.

[B12] Slutsky M, Levin JL, Levy BS (1999). Azoospermia and oligospermia among a large cohort of DBCP applicators in 12 countries. Int J Occup Environ Health.

[B13] Schalie WH van der, Gardner HS, Bantle JA, De Rosa CT, Finch RA, Reif JS, Reuter RH, Backer LC, Burger J, Folmar LC, Stokes WS (1999). Animal as sentinels of human health hazards of environmental chemicals. Environ Health Perspect.

[B14] Rowe PJ, Comhaire FH, Hargreave TB, Mellows HJ (1993). WHO manual for the Standardized Investigation and Diagnosis of the Infertile Couple.

[B15] Bujan L, Daudin M, Charlet JP, Thonneau P, Mieusset R (2000). Increase in scrotal temperature in car drivers. Hum Reprod.

[B16] World Health Organization (1999). WHO laboratory manual for the examination of human semen and semen-cervical mucus interaction.

[B17] Auger J, Eustache F, Andersen AG, Irvine DS, Jorgensen N, Skakkebaek NE, Suominen J, Toppari J, Vierula M, Jouannet P (2001). Sperm morphological defects related to environment, lifestyle and medical history of 1001 male partners of pregnant women from four European cities. Hum Reprod.

[B18] Lorvelec O, Pascal M, Delloue X, Chapuis JL Les mammifères terrestres non volants des Antilles françaises et l'introduction récente d'un écureuil [in French]. Revue d'Écologie (Terre & Vie).

[B19] Lorvelec O, Pascal M (2005). French alien mammal eradication attempts and their consequences on the native fauna and flora. Biological Invasions.

[B20] Bindels JG, Bessems GJ, de man BM, Hoenders HJ (1983). Comparative and age-dependent aspects of crystallin size and distribution in human, rabbit, bovine, rat, chicken, duck, frog and dog lenses. Comp Biochem Physiol B.

[B21] Velez de la Calle JF, Jegou B (1990). Protection by steroid contraceptives against procarbazine-induced sterility and genotoxicity in male rats. Cancer Res.

[B22] Jégou B, Velez de la Calle JF, Bauche F (1991). Protective effect of medroxyprogesterone acetate plus testosterone against radiation-induced damage to the reproductive function of male rats and their offspring. Proc Natl Acad Sci (USA).

[B23] Grenland S, Rothman KJ, Rothman KJ, Greenland S (1998). Introduction to stratified analysis. Modern Epidemiology.

[B24] Cohn WJ, Boylan JJ, Blanke RV, Fariss MW, Howell JR, Guzelian PS (1978). Treatment of chlordecone (Kepone) toxicity with cholestyramine: results of a controlled clinical trial. N Engl J Med.

[B25] Ratcliffe JM, Schrader SM, Steenland K, Clapp DE, Turner T, Hornunq RV (1987). Semen quality in papaya workers with long term exposure to ethylene dibromide. Br J Ind Med.

[B26] Cohn BA, Overstreet JW, Fogel RJ, Brazil CK, Baird DD, Cirillo PM (2002). Epidemiologic studies of human semen quality: considerations for study design. Am J Epidemiol.

[B27] Eustache F, Auger J, Cabrol D, Jouannet P (2004). Are volunteers delivering semen samples in fertility studies a biased population?. Hum Reprod.

[B28] Morris PA (1972). A review of mammalian age determination methods. Mammal Review.

[B29] Pascal M, Damange JP, Douville P, Guédon G (1988). Recherche de critères d'âge chez le Campagnol provençal (*Pitymys duodecimcostatus *[De Selys-Longchamps, 1839]) [in French]. Mammalia.

[B30] Jeong SH, Kim BY, Kang HG, Ku HO, Cho JH (2006). Effect of chlorpyrifos-methyl on steroid and thyroid hormones in rat F0- and F1-generations. Toxicology.

[B31] Bustos-Obregon E, Gonzalez-Hormazabal P (2003). Effect of a single dose of malathion on spermatogenesis in mice. Asian J Androl.

[B32] Abd el-Aziz MI, Sahlab AM, Abd el-Khalik M (1994). Influence of diazinon and deltamethrin on reproductive organs and fertility of male rats. Dtsch Tierarztl Wochenschr.

[B33] Porter WP, Green SM, Debbink NL, Carlson I (1993). Groundwater pesticides: interactive effects of low concentrations of carbamates aldicarb and methomyl and the triazine metribuzin on thyroxine and somatotropin levels in white rats. J Toxicol Environ Health.

[B34] El-Alfy AT, Bernache E, Schlenk D (2002). Gender differences in the effect of salinity on aldicarb uptake, elimination, and in vitro metabolism in Japanese medaka, *Oryzias latipes*. Aquat Toxicol.

[B35] Quénel P (2005). Pesticides organochlorés et santé publique aux Antilles Françaises [in French]. Bulletin d'Alertes et de Surveillance Antilles Guyane.

